# Micro-costing studies in the health and medical literature: protocol for a systematic review

**DOI:** 10.1186/2046-4053-3-47

**Published:** 2014-05-21

**Authors:** Xiao Xu, Holly K Grossetta Nardini, Jennifer Prah Ruger

**Affiliations:** 1 Department of Obstetrics, Gynecology and Reproductive Sciences, Yale University School of Medicine, 310 Cedar Street, LSOG 205B, PO Box 208063, 06520 New Haven, CT, USA; 2 Cushing/Whitney Medical Library, Yale University, 333 Cedar Street, 06510 New Haven, CT, USA; 3 Department of Medical Ethics and Health Policy, Perelman School of Medicine; The Leonard Davis Institute of Health Economics; University of Pennsylvania, 3401 Market Street, 19104 Philadelphia, PA, USA

**Keywords:** Systematic review, Micro-costing, Health, Economic evaluation, Cost analysis

## Abstract

**Background:**

Micro-costing is a cost estimation method that allows for precise assessment of the economic costs of health interventions. It has been demonstrated to be particularly useful for estimating the costs of new interventions, for interventions with large variability across providers, and for estimating the true costs to the health system and to society. However, existing guidelines for economic evaluations do not provide sufficient detail of the methods and techniques to use when conducting micro-costing analyses. Therefore, the purpose of this study is to review the current literature on micro-costing studies of health and medical interventions, strategies, and programs to assess the variation in micro-costing methodology and the quality of existing studies. This will inform current practice in conducting and reporting micro-costing studies and lead to greater standardization in methodology in the future.

**Methods/Design:**

We will perform a systematic review of the current literature on micro-costing studies of health and medical interventions, strategies, and programs. Using rigorously designed search strategies, we will search Ovid MEDLINE, EconLit, BIOSIS Previews, Embase, Scopus, and the National Health Service Economic Evaluation Database (NHS EED) to identify relevant English-language articles. These searches will be supplemented by a review of the references of relevant articles identified. Two members of the review team will independently extract detailed information on the design and characteristics of each included article using a standardized data collection form. A third reviewer will be consulted to resolve discrepancies. We will use checklists that have been developed for critical appraisal of health economics studies to evaluate the quality and potential risk of bias of included studies.

**Discussion:**

This systematic review will provide useful information to help standardize the methods and techniques for conducting and reporting micro-costing studies in research, which can improve the quality and transparency of future studies and enhance comparability and interpretation of findings. In the long run, these efforts will facilitate clinical and health policy decision-making about resource allocation.

**Trial registration:**

Systematic review registration: PROSPERO CRD42014007453.

## Background

Micro-costing is a cost estimation method that involves the ‘direct enumeration and costing out of every input consumed in the treatment of a particular patient’ [1]. In contrast to gross-costing studies that often reflect reimbursement amounts or charges, micro-costing improves precision in cost estimation and reflects actual resource use and economic costs by collecting detailed data on resources utilized and the unit costs of those resources. Unlike gross-costing methods, which estimate average levels and are unable to provide transparent and consistent estimates, micro-costing findings reflect the true costs to the healthcare system and to society. Previous research has shown that using micro-costing methods to measure important cost components helps improve the validity and reliability of total cost estimates for hospital services, and for diagnostic or treatment interventions where costs are not available or evolving [2-5]. It is particularly useful for estimating the costs of new interventions or treatments when there is no established estimate for their aggregate costs [6,7].

The widespread concern about increasing healthcare costs in the USA, as well as in other countries, has prompted growing interest in studying the cost, cost-effectiveness, and cost-benefit of health interventions. It is essential that the costs of health interventions be assessed rigorously in order to inform efficient resource allocation. Cost estimation is the foundation for any economic evaluation. The U.S. Panel on Cost Effectiveness in Health and Medicine has recommended micro-costing as the preferred approach to cost estimation when the alternative gross-costing estimation could cause bias [1]. However, existing guidelines for economic evaluations do not provide sufficient detail for the methods and techniques to use when conducting micro-costing analyses [6].

In this study, we propose to review the current literature on micro-costing studies of health and medical interventions, strategies, and programs to assess how widely and variably micro-costing methodology has been used and the quality of existing studies. Findings from this review will help identify gaps in current application of micro-costing analyses, inform the development of guidelines for micro-costing methodology and a checklist for conducting and reporting micro-costing studies, and ultimately improve the transparency and comparability of future micro-costing studies.

## Methods/Design

The design of this systematic review follows recommendations in the Preferred Reporting Items for Systematic Reviews and Meta-Analyses (PRISMA) statement [8], guidance from the Campbell and Cochrane Economics Methods Group on incorporating economics evidence in systematic reviews [9], and criteria in the Assessment of Multiple Systematic Reviews (AMSTAR) [10]. To help guide the development of the protocol, we conducted a preliminary assessment of the literature using a pilot list of search terms and a single database (PubMed) from March to July 2013. This preliminary assessment helped us identify the terms that authors use when referencing micro-costing methods, the type of health interventions evaluated, the study settings involved, and the types of analysis conducted. Based on results from this preliminary assessment, we refined our search terms, the study selection criteria, the data items to be extracted, and the analytic framework needed to comprehensively evaluate the quality of these studies. Details of our final study protocol are provided below.

### Objective/research question

The purpose of this study is to address the question: How has micro-costing methodology been used in studying health and medical interventions, strategies, and programs? We will take a systematic approach by reviewing micro-costing studies in the health and medical literature to understand both the scope (how widely and variably has this methodology been used) and the quality (how well has this methodology been applied and reported) of these studies. The interventions, strategies and programs may include preventive, diagnostic, and treatment approaches and technologies, as well as public health initiatives.

### Protocol and registration

This systematic review has been registered at the International Prospective Register of Systematic Reviews (PROSPERO) (registration #: CRD42014007453) [11].

### Information sources and search strategy

We will search Ovid MEDLINE, EconLit, BIOSIS Previews, Embase, Scopus, and the National Health Service Economic Evaluation Database (NHS EED) to identify relevant English-language articles. This list of bibliographic databases was determined after careful scoping searches and assessments of the 2011 Health Economic Core Library Recommendations by the U.S. National Library of Medicine [12].

We will use the search strategy outlined in Table 1 for MEDLINE. The search strategies for other databases will be similar but tailored slightly to fit each specific database. Search results from the various databases will be combined and results will be de-duplicated. This will be supplemented by a review of the references of relevant articles identified. Auto-alerts will provide literature updates while the data are being analyzed.

**Table 1:** Search strategy for MEDLINE

	
1.	(microcost$ or micro-cost$).mp.
2.	bottom-up.mp.
3.	“costs and cost analysis”/or health care costs/or health expenditures/
4.	2 and 3
5.	(bottom-up adj5 cost$).mp.
6.	(bottom-up adj5 accounting).mp.
7.	(activity-based adj5 accounting).mp.
8.	(activity-based adj5 cost$).mp.
9.	1 or 4 or 5 or 6 or 7 or 8
10.	limit 9 to english language
11.	limit 10 to journal article

### Eligibility criteria and study selection

We will include studies that meet the following criteria: (1) involve original economic analysis of data; (2) involve a health- or healthcare-related intervention, strategy, or program; and (3) involve the use of micro-costing methodology as defined by Gold *et al.*[1] that involves the ‘direct enumeration and costing out of every input consumed in the treatment of a particular patient’. Economic analysis will include both full economic evaluations and partial economic evaluations, as well as randomized trials or other types of single effectiveness studies that evaluate cost of care [9]. We will operationalize the Gold *et al.*[1] definition for micro-costing by including studies that involve: (1) patient-level data on direct enumeration and costing of all inputs consumed with clear delineation of unit cost information; or (2) program-level direct enumeration and costing of all inputs consumed (with clear delineation of unit cost information) which was then divided across individual participants. Because complete micro-costing may not be feasible in many situations, we will also consider studies that predominantly used micro-costing in their cost estimation. We will exclude studies published only as abstracts, in a language other than English, reflecting biomedical or laboratory research, reporting quantities of resource utilization (for example, length of stay) rather than costs, focusing on identifying cost predictors (for example, analyze impact of patient sociodemographics on cost of care) rather than evaluating costs, or only partially applying the micro-costing methodology (for example, use a combination of micro-costing and gross-costing). We will also exclude reviews, conceptual papers, commentaries, letters, editorials, and papers that only report a study’s methodology without results.

Search results from the various databases will be combined and results will be de-duplicated. A two-phase screening process will be implemented (Table 2). The first phase will screen for full-length, original research articles that involved economic evaluation of a health- or healthcare-related topic. Then, for articles that fulfill the criteria from the first phase screening, we will assess the type of costing methods used and the extent to which micro-costing technique was applied in the cost estimation. This detailed screening will allow us to categorize, summarize, and report reasons for excluded studies. Moreover, we will be able to provide information on the nature of articles that involve some enumeration of resource utilization and unit cost data but do not fully meet the definition of micro-costing methodology [1] (for example, direct enumeration and unit cost data for some inputs consumed plus gross-costing or additional costing methodologies for other cost components).

**Table 2:** Items on article screening form

	
Phase 1 screening:	
	Full length article (yes/no)
	Original research article (yes/no)
	Economic evaluation (yes/no)
	Health- or healthcare-related (yes/no)
	Other reason for exclusion (yes/no, and if yes, specify the reason)
	All phase 1 screening criteria met (yes/no)
Phase 2 screening (if all phase 1 screening criteria are met):	
	Costing methods clear (yes/no)
	Applied gross-costing (entirely gross-costing, partial gross-costing, no gross-costing, or not clear)
	Applied micro-costing (yes, no, or not clear)
	Extent of micro-costing (if micro-costing used): for example, whether micro-costing was used for the entire study or for part of the study
	Type of micro-costing (if micro-costing used): patient-level direct enumeration and unit cost of all inputs consumed; program-level direct enumeration and unit cost of all inputs consumed, then allocated to each participant; and so on.
	Applied other costing methods (yes, no, or not clear, and if yes, specify the methods used)
	Whether authors referred to their study as micro-costing (yes/no)
	Whether to include the article in final data extraction (yes/no)
	Any additional notes about this article

The screening will be based on inspection of study titles, abstracts, and full-text articles. Evaluation of full-text articles will be important because abstracts include only limited descriptions of study design and often cannot provide sufficient detail about a study’s cost valuation methodology. All identified articles from the initial search will be independently reviewed by two members of the review team to determine their appropriateness for inclusion. Any disagreements will be discussed and resolved by consensus. If a disagreement cannot be resolved, a third researcher will be consulted to help resolve disagreement and determine whether the article is suitable for inclusion.

### Data items

For each included article, we will extract detailed information on its characteristics using a standardized data collection form. The International Society for Pharmacoeconomics and Outcomes Research (ISPOR) recently issued the Consolidated Health Economic Evaluation Reporting Standards (CHEERS) guidelines that consolidated and updated prior checklists for reporting of health economic evaluations and are endorsed by 10 biomedical journals [13]. Our data items will be selected based on the CHEERS guidelines [13], guidance from the Campbell and Cochrane Economics Methods Group on incorporating economics evidence in systematic reviews [9], and our previous experience with systematic reviews of health economics studies and with micro-costing studies [5,14-18].

A draft list of data items is presented in Table 3. We specifically tailored this list to suit the aim of this review, that is, to focus on each study’s cost estimation methods. For example, we plan to record whether the study separately reported input utilization quantity and unit cost data which are important for the conduct and interpretation of micro-costing analysis [7], method of quantity data collection used (for example, time-motion study, patient self-report, cost-accounting database, or provider/staff interview), and method of unit cost data collection (for example, invoice amount, or standard fee schedule). Any additional features of a study that deserve consideration when evaluating its quality will be noted in a ‘notes’ data field. We will first pilot a data collection form containing these data items and modify/refine it as needed prior to its final use.

**Table 3:** Items on data collection form for included articles

	
1.	Author
2.	Year of publication
3.	Journal name
4.	Research topic and study questions
5.	Disease category: disease name, disease classification based on International Classification of Diseases, Ninth Revision, Clinical Modification (ICD-9-CM) chapters (for example, infectious and parasitic diseases, neoplasms, diseases of the circulatory system, and so on)
6.	Study intervention(s)
7.	Comparator intervention(s)
8.	Study population/patient characteristics: age, gender, race/ethnicity, inclusion of vulnerable population, and so on
9.	Study setting
10.	Country/jurisdiction
11.	Sample size
12.	Year(s) of study
13.	Study perspective: societal, healthcare system, hospital, healthcare program, and so on
14.	Time horizon
15.	Discounting
16.	Price year
17.	Inflation adjustment
18.	Currency
19.	Currency conversion
20.	Type of economic analysis: cost effectiveness analysis, cost utility analysis, cost benefit analysis, cost minimization analysis, cost comparison analysis, cost outcome description, cost of illness study, and so on
21.	Study type and design: randomized clinical trial, observational study, decision analytic modeling, other economic modeling, and so on
22.	Economic outcome(s)
23.	Health outcome(s)
24.	Methods used to define effectiveness and preferences
25.	Cost components included: personnel costs, consumables/materials/supplies cost, medication cost, facility cost, transportation cost, productivity loss, and so on
26.	Separate reporting of input utilization quantity and unit cost data
27.	Method of quantity data collection: time-motion study, patient self-report, cost-accounting database, provider/staff interview, and so on
28.	Method of unit cost data collection: invoice amount, hospital/clinic/provider price catalogue, national/regional/provincial/hospital/insurer fee schedule, human resources/payroll record, and so on
29.	Study assumption(s)
30.	Sensitivity analyses performed: stochastic (probabilistic) sensitivity analysis, deterministic sensitivity analysis, or no sensitivity analysis
31.	Whether the study referred to its own methodology as micro-costing
32.	Comparison with other economic evaluation
33.	Funding source: industry sponsored study, non-profit funding sources, no funding, or not specified
34.	Conflict of interest: yes, no, or not reported
35.	Notes (please record any additional features of the study that deserve consideration when evaluating its quality)

### Data collection process for included articles

For each included article, two members of the review team will independently extract the data items using the data collection form described above. Disagreements will be discussed and resolved. If disagreements cannot be resolved, a third researcher will be consulted to help determine the most appropriate answer.

### Assessment of study quality and risk of bias in individual studies

In order to assess the quality and potential risk of bias in individual studies, we will critically assess the quality of each included study. This will be performed by using checklists that have been developed for critical appraisal of health economics studies. The Campbell and Cochrane Economics Methods Group [9] has recommended the use of three established checklists for appraising reporting and methodological quality of economic evaluations: (1) the Drummond checklist [19]; (2) the Evers checklist [20]; and (3) the Phillips checklist [21]. A systematic review of quality assessment tools for conducting and reporting health economic evaluations [22] also showed good evidence for inter-rater reliability and test-retest reliability for the Drummond checklist [19] and the Evers checklist [20]. Therefore, we will use the Drummond checklist [19] and the Evers checklist [20] to evaluate the quality and risk of bias for single effectiveness studies, and the Phillips checklist [21] for assessing the quality and risk of bias for studies that relied on decision analytic modeling.

In addition, we will adopt the criteria developed by Fukuda and Immanaka [23] to assess the transparency of cost estimates in included economic evaluations. These criteria categorize studies into transparency levels based on whether each study clarifies what cost components are included, whether the quantity and unit price of resources are separately reported, and whether an estimate of each cost component is reported. This also helps assess the quality and potential risk of bias for each included study. However, no checklist for appraising reporting and methodological quality of micro-costing studies currently exists.

Two members of the review team will independently assess these checklists for each included article with a consensus determined through discussion. If disagreements cannot be resolved, a third researcher will be consulted to help determine the most appropriate answer. Data from these checklists will be reviewed, summarized, and reported.

### Summary measures and synthesis of results

Because we focus on reviewing and evaluating the methodology and reporting quality of included studies, we will not combine the findings across individual studies. Therefore, there will not be a summary measure for this review.

Instead, we will critically assess the methodological and reporting quality of included studies and provide tabulations and narrative summaries. Data items extracted on the screening and data collection forms will be reviewed, categorized, and compared across studies. For included articles, frequency of different methodological features employed among the studies (for example, use of societal perspective, separate reporting of input utilization and unit cost data source, conducting of sensitivity analysis) will be assessed. We will also summarize characteristics of the included studies (for example, type of economic evaluation, year of study). In particular, we will evaluate and report the adoption/application of micro-costing methodology across different disease categories (for example, infectious and parasitic diseases, neoplasms, diseases of the circulatory system).

As recommended by the Campbell and Cochrane Economics Methods Group [9], we will use tabulations and narrative summary to report the study characteristics. Gaps and limitations in the reporting and/or methodology of these studies will be noted and discussed.

### Additional analyses

We will carefully review articles captured in our systematic review that directly compare micro-costing with other cost valuation methods, and appraise their findings. This will allow us to report comparative information on the performance of alternative cost estimation methods, which can inform future research and policy discussion.

### Risk of bias across studies

Risk of bias across studies is particularly relevant when a systematic review combines evidence on treatment effects across multiple studies. However, our review seeks to evaluate the methodological and reporting quality of micro-costing studies, rather than the effect of any particular intervention, and will not combine results across studies. We will include studies across diseases and conditions, and include a wide variety of health and medical interventions, strategies, and programs. Therefore, we expect that the risk of bias across studies (for example, publication bias, selective reporting bias) will have minimal impact on our findings. Moreover, the traditional tools for assessing publication bias (for example, funnel plots) or selective reporting bias (for example, comparing outcomes listed in the methods section with those reported in the results section) have been designed for examining treatment effect of interventions [6], which cannot be applied to our study. Because of these considerations, our assessment of the risk of bias across studies will be based on evaluations of each study’s funding source (for example, industry sponsored study, non-profit funding sources, no funding, or not specified) [24] and the nature of disclosed conflict of interest for the study.

## Discussion

Micro-costing is a cost estimation method that enables precise estimation of economic costs for health interventions. With a growing interest in economic evaluations, we expect to see an increasing number of studies adopting this methodology. Therefore, standardizing the methods and techniques for conducting and reporting a micro-costing analysis is important. If researchers can establish and follow a standardized method for conducting and reporting micro-costing analyses, the quality and transparency of individual studies will be enhanced, and comparability across studies and interpretation of findings will improve. This systematic review will provide useful information to facilitate this effort. Specific guidelines and checklists for conducting and reporting micro-costing studies do not currently exist but can be developed and will help inform clinical and health policy decision-making about resource allocation in the long run.

We recognize that micro-costing has its own limitations [1,6]. It is labor-intensive to collect such detailed utilization and valuation data. The results may not be widely generalizable, as the data may reflect only the practice at selected sites with specific populations. Hence, it is not always feasible or desirable to use micro-costing in economic evaluations. However, prior studies have demonstrated the usefulness and importance of this methodology in certain scenarios (for example, new interventions, extensive variability across providers, and so on) [2-7,17,18] and advances in electronic administrative databases (for example, proprietary cost-accounting data systems) [6] portend greater ease of individual-based data collection in the future. The purpose of this systematic review is to provide a critical assessment of how micro-costing has been used in the current literature. This information can help set research priorities for future economic evaluations and identify opportunities to improve the quality and reporting of future studies when micro-costing is desired or needed.

## Abbreviations

CHEERS: Consolidated Health Economic Evaluation Reporting Standards; ISPOR: International Society for Pharmacoeconomics and Outcomes Research; NHS EED: National Health Service Economic Evaluation Database; NIDA: National Institute on Drug Abuse; NIH: National Institutes of Health; PRISMA: Preferred Reporting Items for Systematic Reviews and Meta-Analyses; PROSPERO: International Prospective Register of Systematic Reviews.

## Competing interests

The authors declare that they have no competing interests.

## Authors’ contributions

XX designed the systematic review and drafted the manuscript. HKGN developed the search strategy for the systematic review and participated in critical revision of the manuscript. JPR conceived of the study concept, participated in and supervised the design of the systematic review, and participated in drafting and critically revising the manuscript. All authors read and approved the final manuscript.

## Authors’ information

XX is an assistant professor at Yale University Department of Obstetrics, Gynecology and Reproductive Sciences. HKGN is a librarian at Yale University Cushing/Whitney Medical Library. JPR is an associate professor at University of Pennsylvania Perelman School of Medicine Department of Medical Ethics and Health Policy and a senior fellow at University of Pennsylvania Leonard Davis Institute of Health Economics.
